# Adaptive leadership overcomes persistence–responsivity trade-off in flocking

**DOI:** 10.1098/rsif.2019.0853

**Published:** 2020-06-10

**Authors:** Boldizsár Balázs, Gábor Vásárhelyi, Tamás Vicsek

**Affiliations:** 1 Eötvös Loránd University, Budapest, Hungary; 2 MTA-ELTE Statistical and Biological Physics Research Group, Budapest, Hungary

**Keywords:** collective motion, hierarchy, self-organizing drones, response theory, collective behaviour, agent-based modelling

## Abstract

The living world is full of cohesive collectives that have evolved to move together with high efficiency. Schools of fish or flocks of birds maintain their global direction despite significant noise perturbing the individuals, yet they are capable of performing abrupt collective turns when relevant agitation alters the state of a few members. Ruling local fluctuations out of global movement leads to persistence and requires overdamped interaction dynamics, while propagating swift turns throughout the group leads to responsivity and requires underdamped interaction dynamics. In this paper we show a way to avoid this conflict by introducing a time-dependent leadership hierarchy that adapts locally to *will*: agents’ intention of changing direction. Integrating our new concept of *will*-based inter-agent behaviour highly enhances the responsivity of standard collective motion models, thus enables breaking out of their former limit, the persistence-responsivity trade-off. We also show that the increased responsivity to environmental cues scales well with growing flock size. Our solution relies on active communication or advanced cognition for the perception of *will*. The incorporation of these into collective motion is a plausible hypothesis in higher order species, while it is a realizable feature for artificial robots, as demonstrated by our swarm of 52 drones.

## 1. Introduction

To understand the concept of persistence–responsivity trade-off in collective motion, one can draw parallels, in terms of the principles, with the fluctuation–dissipation theorem (FDT) [1–3], which limits even nonequilibrium physical systems [4,5] not to be both permanent and susceptible. In its original form FDT establishes a relation between the variability of any quantity in the equilibrium system (fluctuation) and the irreversible response of that quantity when the system is driven out of equilibrium as a result of external excitement (dissipation). Breaking down the equation into single terms: higher/smaller variability equates to a more/less intensive response.

Here we aim to apply this simplified conclusion to the interdisciplinary field of collective motion, where many key notions of traditional statistical physics (e.g. order parameters [6], phase transitions [7], entropy [8,9], gas, liquid and solid [10] or smectic [11] phases, first and second sound modes [12], hydrodynamic regimes [13], broken symmetries [14]) have already found their analogous counterpart.

A properly fitted mathematical formula for FDT in collective motion has been introduced by [15]; however, although it is genuinely reassuring from a statistical physics point of view, the paper has no intention to arrive to any consequence relevant for biological or artificial flocks. Here we approach the question differently. Instead of another rigorous mathematical analogue, we purely concentrate on the core message of FDT. It should be the ‘deep connection between fluctuation and response’, as illustrated by the empirical data in [16].

This deep connection is rooted in the following: when a particle deviates from its previous state as a consequence of either inner fluctuations or some external field, surrounding particles perceive these two processes exactly the same way. Strong response to external fields comes together with strong response to inner fluctuations. On the other hand, reacting scarcely to external perturbations also prevents any local variation to spread across the system globally. Each of these considerations is applicable to collective motion, so it is reasonable to expect that the cardinality of biological units in a system and the presence of noise together force permanence and susceptibility to be reciprocals of each other.

Indeed, as long as collective motion in life can be described by particles interacting in a simple, physics-type manner, i.e. taking on an orientation according to their neighbouring conspecifics [17,18], the corresponding systems exhibit a few characteristic patterns of motion. Some of these are simple flows in colonies of cells [19–22], while other forms are reminiscent of turbulence [23–25]. The fact that the governing forces of such systems are more complex than simple electromagnetic forces (such as those in systems in traditional statistical physics) does not resolve the conflict: these systems are bound to choose either high persistence or high responsivity when they exhibit collective motion. As both features are desirable this situation is referred to as the *persistence–responsivity trade-off* .

The tight vicinity of a critical point–where phase transitions occur–provides any system a chance to bypass the trade-off. Here the magnitude of order rises above zero, while the susceptibility to external fields decreases from infinity. A collectively moving group can exploit the simultaneous presence of order and susceptibility if it fine-tunes its internal parameters—and, hence, its stability—in a way that the system’s critical noise level is raised just above the given external noise. With the presumption of global adaptation to noise, criticality typically causes correlations to become scale-free and thus a significant degree of instantaneous order emerges that can also be changed at any time [24,26]. However, the emerging global velocity direction also becomes too easily changed by the near-critical noise, hence it does not prevail.

In contrast, for higher order species, such as birds [27] or ungulates [28], collective motion not only means *persistent* movement of the masses on long time scales, but also a rapid and global *response* to external stimuli, hence exploiting the advantage of having many eyes, increasing the overall vigilance of the surrounding world [29,30] if opacity is not too high [31]. The question of *how* these systems achieve this simultaneously agile and reliable behaviour arises naturally. In the simplest models of flocking the information which is shared by the agents is their momentary position and velocity; future positions and velocities are determined only from these inputs. Over the years several improvements have been introduced concerning various further aspects of the interactions. One of the relevant features introduced by Zhang *et al.* [32,33] was taking into account the predictions that the members of a flock can make concerning the expected trajectories of their neighbours. In addition to their theoretical results, these authors showed that their algorithm based on predictions led to a consensus (common direction) being reached rapidly in the systems they studied (mostly fully connected with weighted links, moderate size and no boundaries).

Our main assumption is that the members of a group are able to communicate information beyond their momentary position and velocity. This ability of the flocking agents is both obvious from observations and has been addressed in other studies. In groups of animals there are several possible ways by which an individual can send a signal to the rest of the group. A sudden change in the orientation or a specific vocalization may serve as cues. Strandburg-Peshkin *et al.* [34] investigated the effects of visual sensing among flock mates, resulting (owing to the associated limitations) in a representation of the state of the flock that was different from the actual geometry. A few studies have addressed the question of escape waves, which clearly influence the behaviour of a flock as a whole. For example, in [35,36] it was discussed how a local perturbation due to predatory attack may result in a wave travelling over the whole flock. In this approach the sudden change in velocity in a given part of the flock serves as a cue for the whole group, thus acting on a global scale. In addition, even the position of a given individual (e.g. being in front of the flock) can have a specific signalling role for the rest of the flock-mates [37]. In the present work, however, we introduce a way of taking into account cues using a model in which the positions and the velocities of the flock members are updated by taking into account the information from some specific flock members (whose role changes with time) conveyed through means other than simply changing position or velocity.

To achieve this, individuals are driven to develop cognitive capabilities that are often termed ‘theory of mind’, especially in the case of primates [38]. This enables agents to perceive their social environment and to distinguish between movements that are interpreted as intentional and those that are not, so that changes induced by external information, such as predators or obstacles, can be transmitted across the collective with underdamped propagation, while distortions of ever-present noise can be ruled out with overdamped dynamics. This sets up the stage for consensus finding [39–42], which is a pre-requisite for flocking, a spectacular manifestation of collective behaviour that gained attention in the context of collective robotics as well [43–45].

From early on [6,10,13,17] most of the experimental and modelling approaches assumed egalitarian interactions to describe flocking and schooling [8,23,46–48]. However, when the question of leadership was raised by Couzin *et al.* [49], the science community found increasingly complex internal organization principles during group motion [50–52]. Combination of two of the most widespread behavioural phenomena of collectives, namely flocking and hierarchies, seems almost inevitable. Hierarchical structures are prevalent from nature to society [53], because they can be shown to result in more efficient group performance [54–56] by optimizing the flow of information in systems. This feature is the reason we incorporate hierarchy into the new models presented in this paper.

To demonstrate how persistence and responsivity can be achieved simultaneously, resulting in efficient flocking even in complex environments, we investigate some of the most commonly used agent-based models of collective motion enhanced with a mathematically simple way of adapting the interaction network hierarchically. We introduce agents of a new kind that take account of more than the position and velocity of flock-mates. Through communication signs, or by perceiving certain minor changes in physical phase space as cues, they gain knowledge on the intention, i.e. *will*, of their neighbours as well. Based on this *will* state—which is attributed to agents possessing globally relevant information—local neighbours change their interaction network, declare agents with intention as local leaders (centres of a locally star-like leadership hierarchy) and direct their motion, and thus the motion of the collective in response to this intention, with high efficiency.

Going beyond the prior paradigm of time-independent leader–follower relationships, here we no longer assume that inner states and the leader–follower behavioural patterns are irrespective of the actual situation of the flock. Instead, the role an agent plays changes in accordance with the changing situations in such a way that collective performance (ability to be both persistent and responsive) becomes close to optimal.

After defining the models we create pure, isolated scenarios in a simulation in which we can clearly demonstrate that agents with this extra information transmission can indeed enhance their responsivity and thus break out of the previous trade-off with unprecedented efficiency: they move steadily in the long term and yet make rapid and accurate global shifts as a correct reaction to the environment. The final section demonstrates that this finding on the level of principles easily translates to practical developments by surpassing previous solutions of moving large numbers of artificial agents in a closed space with high speed and coherence.

## 2. Model definitions

We investigate several agent-based models of collective motion with simulations in two dimensions. First, we temporarily omit the interaction terms that maintain the metric distance of neighbours (repulsion and attraction), and focus on the only remaining interaction term, the velocity alignment, which essentially determines how the flock maintains or changes its global direction.

Instead of metric distance, we choose the *N*_*i*_ neighbourhood of agent *i* on a topological basis, meaning that every agent interacts with its *k* nearest neighbours (and self).

Besides the empirical evidence that the topological neighbourhood describes some real flocks better [57] there is another reason for this choice. In a topological neighbourhood interactions cannot break down owing to spatial separation, so one does not have to deal with the spatial diffusion of the flock's shape in this special casewhere attraction is not keeping agents together (besides, diffusion is negligible in all our simulations, even on the longest time scale investigated).

To analyse the differences in the models, we present them in an easily comparable form. We always express the desired velocity of agents as $v_{i}^{\mathrm{desired}}=v_{0}{\hat{v}}_{i}$, where *v*_0_ is a constant flocking speed and ${\hat{v}}_{i}$ is the desired direction of individual motion. In the following sections, the equations for these desired velocities are detailed and compared.

### 2.1. Original self-propelled particle model

The first model we analyse in this paper is the standard Vicsek model [6]; hereinafter, the *ViSt* model. Here we use the notation ViSt for the case (equation (2.1)) in which only the central idea of the updating rule of the original model—i.e. averaging over the directions of the neighbours—is accounted for,

2.1 $${\hat{v}}_{i}^{\mathrm{ViSt}}(t+\delta t)=N\left(\sum_{\;j\in N_{i}}v_{j}(t)\right),$$

where **v**_*j*_(*t*) is the velocity vector of agent *j* at time *t* and N(v)=v/|v| is the normalization operator of a vector. Additionally, in the simulations above and in the rest of the models, a relatively low noise level is used (to be specified later).

### 2.2. Couzin leader model

An interesting and simple extension of the ViSt model is the Couzin leader model [49]; hereinafter, the *CoLd* model. The CoLd model was introduced to investigate how informed (leader) agents can drive a flock in a desired direction. It extends the ViSt model by assigning a continuous *will* factor *w*_*i*_ to agents, which is zero for followers and 1 for leaders who tend to follow their desired direction ($\hat{d}$) instead of the local average,

2.2 $${\hat{v}}_{i}^{\mathrm{CoLd}}(t+\delta t)=N((1-w_{i}){\hat{v}}_{i}^{\mathrm{ViSt}}+w_{i}{\hat{d}}_{i}).$$

**Figure 1.**
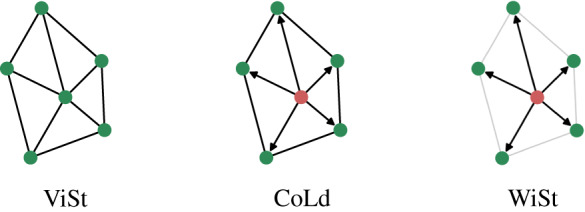
Visual representation of how the desired velocity of the central agent is calculated in three different ways, corresponding to the ViSt, CoLd and WiSt models. In the ViSt model every agent is allocated the same weight in the local velocity average, thus the interactions are symmetric (mutual links). In the CoLd model the symmetry breaks (directed links) as the informed (red) agent tends not to listen to its neighbours, but the others still do not differentiate between the informed and other neighbours in their local velocity average. In the WiSt model the uninformed neighbours are aware of the informed state of the red agent, so they also lower the weights (grey links) of every other uninformed neighbour in their local average, which are dominated by the velocity of the informed agent.

Note that an agent’s new velocity is *only affected by its own will*, as illustrated by figure 1.

### 2.3. Spin model of the Cavagna group

The third model we investigate is the inertial spin model proposed by Cavagna *et al.* [58]; hereinafter, the *CaSp* model. This elegant model describes collectively moving particles based on symmetry arguments. The particle physics approach used is based on the observation that collectively moving animals keep moving at a more or less constant speed. Their velocities can thus be described solely by their direction of circular symmetry; therefore, a Hamiltonian-type description of the system must depend on the generator of rotations in the velocity: the spin. The spin is mathematically the same object in constant velocity motion as the angular momentum in constant radius motion (i.e. rotation). Thus, turning flocks are found to be analogous to isotropic antiferromagnets and superfluids.

The translation of this model into the forms of expression in this paper is as follows:

2.3 $${\hat{v}}_{i}^{\mathrm{CaSp}}(t+\delta t)=N(R[v_{i}(t),s_{i}^{\mathrm{ViSt}}(t)\delta t]),$$

where R[v;θ] is the operator that rotates vector **v** at angle *θ* on the *x–y* plane. The spin $s_{i}^{\mathrm{ViSt}}$ tries to align the current velocity to the ViSt velocity by its evolution,

2.4 $$s_{i}^{\mathrm{ViSt}}(t+\delta t)=s_{i}^{\mathrm{ViSt}}(t)\;{\text{e}}^{-(\delta t/\tau )}+J\left[\frac{v_{i}(t)}{v_{0}}\times {\hat{v}}_{i}^{\mathrm{ViSt}}\right]\cdot {\hat{e}}_{z}\delta t,$$

where ${\hat{e}}_{z}$ is the unit vector along the *z* axis. Equation (2.4) works as follows: if there is a misalignment between the velocity of the agent, **v**_*i*_, and the velocity subject to alignment by the spinning mechanism (here ${\hat{v}}_{i}^{\mathrm{ViSt}}$) then the spin increases and, through equation (2.3), aligns the individual velocity to the local average. Here two free parameters are implemented (see electronic supplementary material, text S1 for their relation to the original model): *τ*, the characteristic lifetime of the *s* spin of the agents, and *J*, the coupling strength between the velocity differences and the spin. Different parameter choices can lead either back to the overdamped limit of the ViSt model (*τ* → 0) or to an underdamped phase (*τ* > size of flock/speed of spin-wave), where spin waves can travel through the flock and enable collective turns with equal radii.

### 2.4. New models with *will* recognition

The two models we introduce are analogous extensions of the CoLd and CaSp models. The essence of the new behavioural rule in both new models is that—owing to some kind of cognitive capability—agents are aware of the level of determination (*will*) of their neighbours. This can be imagined in various ways, such as: (i) attention to active signalling (e.g. use of brake lights), (ii) recognition of unusual behaviour (e.g. reaction to predators), or (iii) differentiation between noise-induced and purposeful manoeuvres (e.g. trained cognitive capabilities to analyse the fine motion of neighbours).

In our models, every agent *j* has a momentary *will* factor *w*_*j*_ with a value of [0, 1] (time dependence becomes relevant later; see §3.5). The *will* factor is a representation of new or important information an agent possesses and this determines how much influence an agent has on its own velocity and—as a crucial improvement to *w*_*j*_ used in the CoLd model—*also on the velocities of its neighbours*. As illustrated in figure 1, this approach leads to a temporal local star-shaped hierarchy dominated by informed agents. Note that star structures are considered to be maximally hierarchical according to most graph theoretical measures (e.g. [59]).

Our first new model (called the standard *will* model, referred to as WiSt from now on), the extension of the ViSt/CoLd models, takes the following form:

2.5 $${\hat{v}}_{i}^{\mathrm{WiSt}}(t+\delta t)=N\left({\hat{v}}_{i}^{\mathrm{CoLd}}+\sum_{\;j\in N_{i}}w_{j}\frac{v_{j}(t)}{v_{0}}\right).$$

Finally, we merge our *will*-based approach with the original CaSp model. In the CaSp model agents align to the ViSt velocity through rotations. ViSt velocity extended by the enhanced influence of informed agents is the WiSt velocity, so the natural extension of the CaSp model with *will* recognition is aligned through the spinning mechanism to WiSt velocity. The resulting *will* + spin, or simply WiSp model, is the last model to be investigated and compared,

2.6 $${\hat{v}}_{i}^{\mathrm{WiSp}}(t+\delta t)=N(R[v_{i}(t),s_{i}^{\mathrm{WiSp}}(t)\delta t]),$$

where, in accordance with equation (2.4), the evolution of the spin towards the *will*-weighted velocity is

2.7 $$s_{i}^{\mathrm{WiSp}}(t+\delta t)=s_{i}^{\mathrm{WiSp}}(t)\;{\text{e}}^{-(\delta t/\tau )}+J\left[\frac{v_{i}(t)}{v_{0}}\times {\hat{v}}_{i}^{\mathrm{WiSt}}\right]\cdot {\hat{e}}_{z}\delta t.$$

Equations (2.1), (2.3), (2.5) and (2.6) define the momentary (time dependent) desired velocity an agent tries to reach. The way in which this desired velocity is approximated is determined by the dynamic model of the agent and the physical environment; namely, by some kind of inertia and noise in our simplified models. This results in the following equation for the acceleration of agents (using the same simulation framework as in [43,60]):

2.8 $$a_{i}=N(v_{i}^{\mathrm{desired}}-v_{i})\cdot \min\left(\frac{\left|v_{i}^{\mathrm{desired}}-v_{i}\right|}{\tau_{c}},a_{\max}\right)+\xi ,$$

where *τ*_*c*_ is the characteristic time of exponential convergence to the desired velocity, *a*_max_ is the maximum acceleration of the agents and *ξ* is white noise, a vector drawn from a two-dimensional normal distribution with an expected null vector and variance $\sigma_{\xi }^{2}$. For values during the simulation, see electronic supplementary material, table S1.

## 3. Simulation results

In this section all four models described by equations (2.1), (2.3) and (2.5), (2.6) are investigated from two distinct perspectives, as test beds for measuring responsivity and persistence: (i) how quickly and how accurately a group of agents can change to any new direction if the new information about the desired direction is given only to a single agent; (ii) how long a group of agents can maintain the direction of global movement in the absence of any new information or external threat. To focus purely on the outcome of the tests described above, the effects of stochasticity are minimized first: (i) noise levels are defined to allow all models to maintain a highly polarized (approx. 0.98) collective motion; (ii) every simulation is started with an ordered flock in terms of both position and velocity: *N* agents are placed homogeneously within a circle, separated by 15 m on average, and they all move in the same direction upon release with speed *v*_0_ = 8 m s^−1^.

### 3.1. Quantifying responsivity

In real flocks the global direction of motion changes many times as a result of observed external stimuli, such as obstacles or predators. Here we want to evaluate the collective response regardless of what triggers it. After allowing the noise to cause slight disarray in the initially uniform and perfectly coherent motion for *t*_*s*_ = 3 s, we turn agent 0—initially placed in the centre of the entire flock—with an angle of deviation φ relative to the initial direction. Following this, the desired velocity of agent 0 will always point towards this new direction **n**_φ_, and its *will* in the new models also jumps to and remains at 1,

3.1 $${\hat{v}}_{\mathrm{agent}0}(t>t_{s})={\hat{n}}_{\varphi }.$$

The level of the collective response to this new information is given by the time average of the scalar product of the new direction, **n**_φ_, and the global velocity, $V(t)=1/N\sum_{i}v_{i}(t)$, over a response transient time, *t*_*R*_,

3.2 $$r(\varphi )=\frac{1}{v_{0}t_{R}}\int_{t_{s}}^{t_{s}+t_{R}}V(t)\cdot {\hat{n}}_{\varphi }dt.$$

Typical deviation-angle dependencies can be found in electronic supplementary material, figure S1.

To characterize the general ability of the flock to respond to external stimuli, one should condense the responses depending on the angle into a single descriptive value. The simplest assumption (in the absence of external symmetry breaking) is that the distribution of the desired new global direction is isotropic; thus, we perform multiple tests at different deviation angles and average over them to get the responsivity,

3.3 $$R=\frac{1}{\pi }\int_{0}^{\pi }\langle r(\varphi )\rangle \;\text{d}\varphi ,$$

where 〈 · 〉 symbolizes the average over numerous simulations. In the case of this responsivity test, five simulations are run for each deviation angle. Discretization of the angular integral is 10°.

Note that the values of *R* are mathematically bound between − 1 and 1, but practically between 0 and 1: a flock that keeps on moving straight (**n**_0_) irrespective of agent 0’s changed direction has 0 responsivity, while the perfectly responsive flock that globally copies the velocity of agent 0 at the moment it turns reaches *R* = 1.

Keeping *t*_*R*_ independent from φ is simple and is observed in nature [61]; however, the choice of this value is not obvious. We need to provide the flock with enough time to reach the new velocity direction and then also to exploit high **V** · **n**_φ_ in the time average, but *t_R_* must not be too long in order to see the difference between slow and rapid turns in *R*. The flock spatial size scales with $\sqrt{N}$ in two dimensions, so if we expect linear information propagation, then $t_{R}∼\sqrt{N}$ must hold for comparable results throughout changing flock sizes. For the sake of simplicity, we choose the constant that connects the response time and the square root of flock size to be 1 s, meaning that we measure the turn of a flock of 100 agents for 10 s, which—based on simulations—provided us with the relevant information. This is also approximately the amount of time that an agent in the middle would need to cross the flock, so it is a reasonable time scale for comparison (e.g. [62]).

### 3.2. Responsivity scaling

**Figure 2.**
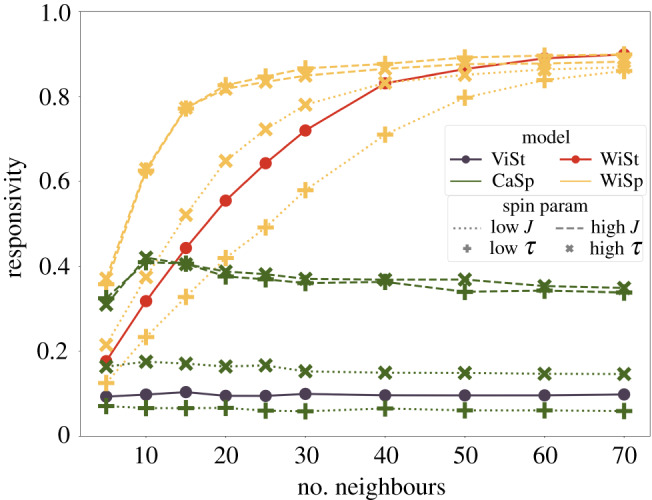
Responsivity as a function of the number of topological neighbours. Colours represent model types: ViSt, dark purple; CaSp, green; WiSt, red; WiSp, gold. In the case of the CaSp and WiSp models, dashed lines correspond to high coupling (*J* = 1400) and dotted lines to low coupling (*J* = 200), while ‘ + ’ corresponds to models where spin dies out quickly (*τ* = 0.3 s) and ‘× ’ to where spin stays in the system longer (*τ* = 0.8 s). Models with *will* recognition (WiSt, WiSp) can always improve their responsivity further by increasing the connectedness of agents, while models that lack the cognitive feature of *will* recognition do not improve in terms of responsivity as *k* grows. Simulations were performed with *N* = 250 agents, *t*_*R*_ = 15.8 s, $\sigma_{\xi }^{2}=1$ m^2^ s^−4^.

First, we investigate how the number of topological neighbours *k* affects the responsivity of a flock following different alignment rules: ViSt, CaSp, WiSt and WiSp. (With a single agent forced to alter direction there is no difference between the ViSt and CoLd models. Since the descriptive power of the CoLd model is apparent when several agents possess information, these responsivity measurements are referred to as ViSt throughout the paper.) As shown in figure 2, the ViSt model has low responsivity because, in low *k* cases, the information diffuses slowly on the sparsely connected interaction network; as *k* increases, the voice of a single turning agent is lost even in the local average. One can also see that the responsivity of the CaSp model is mainly determined by the coupling strength *J*, the parameter that shapes how much spin is injected into the system by a turning agent. Stronger coupling means better responsivity. This observation is in agreement with the idea of a spin being a conservative quantity [58]. To a smaller degree, responsivity is also affected by the lifetime of spin, *τ*. The more agents the spin reaches before it dies out, the more responsive the flock becomes.

Adding *will* recognition to either the ViSt or CaSp models as seen in equations (2.5) and (2.7) enhances the responsivity by a considerable amount—as expected. It is very encouraging that for *k* ≥ 15 even the less responsive new WiSt model is better than all CaSp models in terms of responsivity, notwithstanding the fact that spin models were originally designed to describe collective response (figure 2).

**Figure 3.**
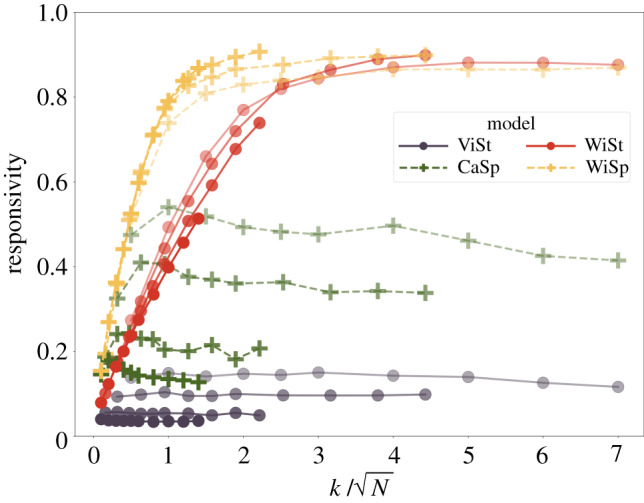
Responsivity as a function of neighbours over the square root of flock size. Colours represent model types: ViSt, dark purple; CaSp, green; WiSt, red; WiSp, gold. Increasing transparency corresponds to decreasing flock size. Models with *will* recognition (WiSt, WiSp) can keep up with increasing flock size by increasing connectedness and they converge to a universal curve while models lacking the cognitive feature of recognizing each other's intention (ViSt, CaSp) lose their responsivity with growing flock size even if they are tuned to be responsive from the outset. Simulations were performed with 100, 250, 1000 and 2500 agents and *t*_*R*_ = 10, 15.8, 31.6, 50 s, respectively. Noise: $\sigma_{\xi }^{2}=1$ m^2^ s^−4^.

Scalability is an essential requirement of collective behaviour in both nature and technology. To compare the models with and without *will* recognition, we studied how the size of the flock (*N*) affects the responsivity of the collective (figure 3). We found that responsivity decreases gradually as the size of the flock increases in the case of models without *will* recognition—irrespective of whether they start from high (CaSp) or low (ViSt) responsivity for small flocks. Meanwhile the same models upgraded with *will* recognition can keep up with increases in the size of the flock if the agents extend their attention to more neighbours. It is very advantageous that, in order to keep the same level of responsivity, the neighbour number, *k*, only needs to scale with $\sqrt{N}$, as seen in figure 3. The relation is not rigorous, as saturation curves of the WiSt model decrease slightly with growing flock size, but curves of the WiSp model seem to converge similarly. It is worth mentioning that the WiSp model in biologically realistic $k/\sqrt{N}≃1$ scenarios has an almost twofold advantage over any other model investigated in terms of responsivity; furthermore, it reaches an impressively high responsivity value of around 0.8. A visual demonstration of the performance of all the models can be found in electronic supplementary material, video S1.

Benchmark studies [8] of collective motion imply that the assumption *k* = 7 corresponds to the highest entropy for the high-resolution data collected from swirling flocks of starlings of different sizes. This means that the simplest interaction network that is already capable of explaining the observations is the one with seven topological neighbours. Our results do not contradict this seminal conclusion. The CaSp and WiSp models have a responsivity—interpolated to *k* = 7—of no lower than around 0.2 even for 2500 agents. The overall value is mainly decreased by rather sharp turns (greater than 120°), which are much more relevant for artificial systems. The global turns recorded for natural systems of starlings typically do not reach this value; they tend to stay in the moderate region of direction change, where models with responsivity of around 0.2 continue to perform well.

### 3.3. Quantifying persistence

It is desirable not only to change the global velocity quickly when needed, but also to keep the global direction stable for a long time when there is no need for a change. To measure how the different models perform from this point of view, we introduce a new measure of persistence. The angle *Φ* between the initial direction of the flock and the momentary global velocity **V** behaves as a random walker if the polarization (*V*/*v*_0_) remains in the high regime. To understand this, imagine the noise as a random perturbation that changes the global velocity slightly. This means that every agent will have approximately this new velocity, and further perturbations will act upon the new direction either by changing it back towards the original direction or by moving it even further away from the initial direction. This is a diffusion process of the angle of the global velocity, which can be described by

3.4 $$\langle {\left(\Delta \Phi \right)}^{2}\rangle =D_{\Phi }t.$$

The diffusion coefficient, *D*_*Φ*_, describes the intensity of the diffusion process. Since agents are disturbed by the same level of noise and they follow the same rules, having *N* agents in a system at a given time instead of a single one is like having *N* times more memory, which results in an *N* times slower time scale in the diffusion process. For a summary of memory in collective motion, see [63]. So, as a baseline, we compare the diffusion coefficient of the global velocity *D*_*Φ*_ for *N* agents with the diffusion coefficient of the velocity of a single particle, disturbed by the same noise with an *N* times slower time scale $D_{\Phi }^{s}/N$. The ratio of these two diffusion processes measures the persistence *P* of a model,

3.5 $$P=\frac{D_{\Phi }^{s}/N}{D_{\Phi }}.$$

Note that *P* is not bounded to be lower than 1, as $D_{\Phi }^{s}/N$ is not a theoretical limit of the diffusion coefficient—it is only a reasonable subject of comparison. Note that it is also the diffusion coefficient of a ViSt model with all possible pairwise interactions (*k* = *N*), where every agent moves towards the global average velocity: $D_{\Phi }^{s}/N=D_{\Phi }^{\mathrm{ViSt}}{|}_{k=N}$. A low diffusion coefficient *D*_*Φ*_ means slow diffusion, which means that the flock can keep its original global velocity while maintaining high polarization for longer times. This is high *persistence*.

### 3.4. Persistence–responsivity trade-off

**Figure 4.**
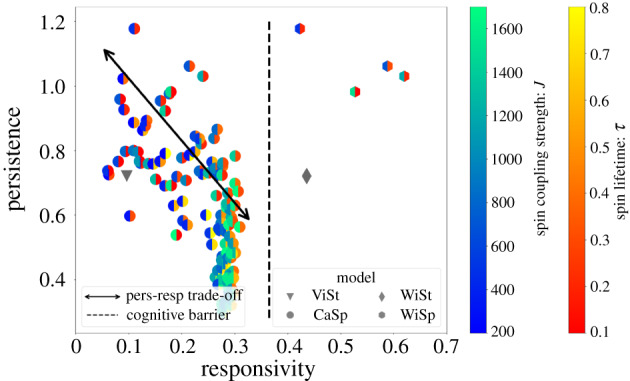
The persistence–responsivity plane. Markers represent model types: inverted triangles, ViSt; circles, CaSp; diamonds, WiSt; hexagons, WiSp. Colours represent the parameters of spin-based models according to the colourbars. The trade-off between persistence and responsivity (double-headed arrow) bounds models consisting of particles without advanced cognitive capabilities. Models with *will* recognition can improve responsivity while maintaining persistence. Such collectives break through the symbolic cognitive barrier (dashed line) and break out of the trade-off. Simulations were performed for *N* = 250 agents, *k* = 15 neighbours, five times for a response time of *t*_*R*_ = 15.8 s and 40 times for a sampling time for persistence of *t*_*P*_ = 600 s. Since the importance of both persistence and responsivity is stressed at high noise, in these simulations noise is doubled relative to previous simulations: $\sigma_{\xi }^{2}=2$ m^2^ s^−4^.

In the spirit of the fluctuation–dissipation theorem, responsivity and persistence are contradicting features of a model. The motion of responsive flocks often strongly fluctuates because of agitation by noise, while persistent movement requires changes in global velocity to be prevented. As long as noise-induced and intentional changes are not separated by individual cognition, there is a trade-off between persistence and responsivity, as shown on the left side of figure 4, where traditional models reside. The ViSt model and likewise underdamped CaSp models (parametrized with low *J* and low *τ*) are highly persistent, but possess low responsivity. In contrast, there are responsive CaSp models with high coupling and long-living spins, but they exhibit unreliably diffusive motion (low persistence). There are intermediate parameters, but these are also bound by the threshold of the persistence–responsivity trade-off, which is visualized in figure 4 as well. The CaSp model is designed to be unifying, hence very rich in behavioural patterns, so it is reasonable to expect that if this model cannot break out of the trade-off, then any other model constrained to the same information about the neighbours will also fail to do so.

In contrast, our new models with *will* recognition, on the right-hand side of the symbolic cognitive barrier shown in figure 4, avoid the previous persistence–responsivity trade-off and provide superior solutions with maintained persistence and highly increased responsivity. A notable achievement of the WiSp model is that, with correctly chosen low *τ* and low *J* parameters, it can outperform any parameter combination of models lacking *will* recognition in terms of *both* persistence and responsivity. Since no new information is injected into the system in any persistence measurement, WiSp always has the same persistence as the CaSp model with the same spin parameters. So with the parameters that lead to the most persistent motion at the cost of low responsivity in the CaSp model, the WiSp model retains its highest persistence, and its responsivity, which is increased by *will*-based hierarchy, surpasses even the most responsive models without cognition. *The collective breaks out of the persistence–responsivity trade-off with the combined power of individual cognitive abilities and adaptive local leadership hierarchy*.

Also with a choice of certain intermediate *τ* and intermediate *J* parameters the collective can reach an impressively high responsivity of ∼0.6. Note that the environment is set deliberately noisy, and still such high responsivity can be achieved with persistence remaining above 1, thus it is even more persistent than the mean-field ViSt model.

### 3.5. Moving in confined environments

After the successful tests of responsivity to a single particle with *will* and persistence in boundless empty space, the concept of *will* is to be tested in a complex use case requiring the simultaneous presence of both persistence and responsivity. Agents are tasked to flock around coherently in a closed arena without collisions. Walls trigger the frontal agents to have a *will* and thus induce a global velocity change. The additional complexity of this scenario compared with isolated unit tests is that (i) there can be any number of agents possessing *will* simultaneously; (ii) desired changes can contradict each other at the corners.

In terms of the WiSp model, the *will* of an agent jumps to 1 when it is forced to turn because of the wall. Two practical questions arise when implementing *will* in such situations, as follows.

*When do walls force agents to turn?* All four linear wall segments of the square-shaped arena have the same virtual width: *R*^Wall^. If an agent gets closer to a wall segment than this threshold, a prohibited angular domain is created for its desired velocity. The centre of the angular domain points perpendicularly towards the wall segment. The size of the angular domain grows linearly starting from 0 at *R*^Wall^ distance from the wall segment and reaches 2*π* exactly at the wall. The overall set of wall segments generates a union of prohibited angular domains. If the desired velocity of the flocking terms defined in equation (3.6) dictates a velocity direction that is inside this unified prohibited domain, the agent turns away to point its desired velocity outside of the domain.

*How do agents turn at walls?* Ideally, every agent in the flock has to be synchronized in terms of turning choices at the wall to stay together. If the flock already started a right-hand turn, every other agent that would later be forced to turn at the wall has to turn right as well. In order to do this, agents see each other’s *will* in a signed form (our convention is + for left turns and − for right ones). When an agent is forced to turn, it sums up the *will* of its neighbours to choose a new velocity direction for the next time step. It chooses the left/right edge of the unified prohibited angular domain according to the sign of the sum. In the absence of *will*ed neighbours, the agent chooses the edge which requires a smaller rotation relative to the rejected velocity given by the interagent social interactions of equation (3.6). The absolute value of the *will* jumps to 1 with a sign in accordance with the direction of its turn. *Will* values decay exponentially with a characteristic lifetime of *τ*_*W*_. The value of this parameter needs to approximate the time that new information remains globally relevant and, hence, is worth spreading.

So far we have introduced the sign of *will* to encode the direction of change, which is seen by others. In the case of a flock of birds, knowledge about the direction of change is relatively easy to access because birds tilt their bodies [36]. If the mere mechanics of a manoeuvre does not require such an apparent sign, efficiency will demand it.

Agents reaching the wall turn the flock effectively by the high absolute value of *will* and the rapid propagation of spin. But there are still some other agents reaching the wall. Since they tend to be neighbours of the first-at-the-wall agent, the sign of *will* ensures that they also start a turn in the same direction as their frontal neighbours. Assuming that everything works well, by the time another agent at a more distant part of the flock encounters another wall, the flock will have turned enough so that its default choice of direction (smaller rotation of desired velocity) will be the continuation of the previous collective turn even if this new agent does not see any *will*ed agent around itself. This self-replicating trend of choice is the key to moving around in unison.

In order to make that happen we introduce the following algorithm (WillFull model) for the desired velocity:

3.6 $$\begin{array}{rl} & {\hat{v}}_{i}^{\mathrm{WillFull}}(t+\delta t)\\ & \;=N[(1-\Omega_{i})R[v_{i}(t);s_{i}^{\mathrm{WiSt}}(t)\delta t]+\Omega_{i}v_{0}{\hat{v}}_{i}^{\mathrm{WiSt}}], \end{array}$$

where $\Omega_{i}=\max(|w_{i}|,\sum_{\;j\in N_{i}}|w_{j}|/\left(1+\sum_{\;j\in N_{i}}|w_{j}|)\right)$.

This model aims to merge the benefits of all models presented so far. It uses the CoLd model scheme for differentiation between (rare) *will*-aware and (abundant) ignorant agents. The majority propagate information through the CaSp model’s underdamped wave transmission of spin, which aligns agents indirectly. But initiators of the waves are *will*ed agents, who find consensus among themselves reliably by applying the overdamped dynamics and direct alignment of the ViSt model. Un*will*ed agents with significant *will* in their neighbourhood do not bother with spin, but follow the leaders they see directly. *Will* recognition amplifies new information *if it is present*, hence it realizes a very responsive flock without losing the ability to maintain persistive motion.

The simulation environment being used now is more realistic in terms of noise level and constraints in communication and motion dynamics. Both are inherited from [43]. Besides making the task and the environment more realistic and thus substantially more challenging, expectations towards the proper behaviour are also raised compared to previous unit tests. Coherent flocking is also expected to be free from both wall–agent and interagent collisions. Therefore, customary terms are added to equation (3.6): repulsion and friction, to avoid collisions and oscillations, respectively, as in [43]. The parameters of the model, including *τ*_*W*_ , *J* and *τ*_*S*_, are optimized using the same evolutionary algorithm as in [43], and are listed in electronic supplementary material, table S2. Note that the rectangular arena acts as an implicit attraction as it does not allow agents to disperse.

Efficient flocking means more than high polarization. From the perspective of artificial agents (robots, drones) it also means other features that natural flocks exhibit: staying together, keeping the average density high, but avoiding dangerously dense packing even during the inevitable turns. To measure the performance of the WillFull model, we simulate a flock of 500 agents starting from the middle of an arena of 1400 × 1400 m, with initial nearest neighbour distance of 20 m. The arena is sliced into a 28 × 28 grid (50 × 50 m each), and in every time step of 0.01 s the population of each grid is counted. The number of agents in the most populated grid represents the maximum density, while the number of agents over the number of populated grids represents the average density. Hence, the expectation towards efficient flocking is that the ratio of maximal and average densities in space remains low and stable in time, meaning that free flight parts of the flock remain dense but density does not peak any higher anywhere either when the need for direction change puts the system under stress.

**Figure 5.**
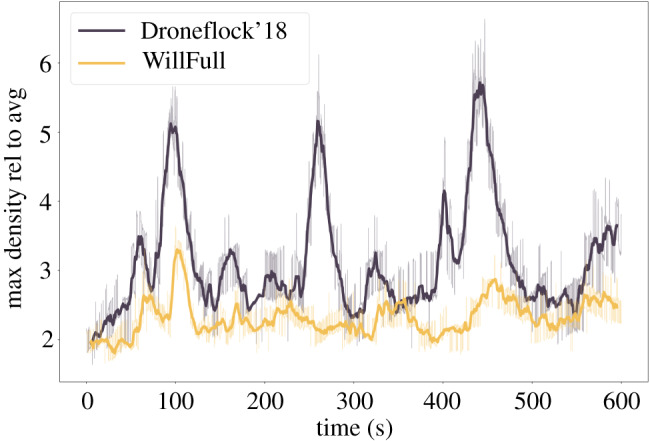
Relative maximum density of simulated flocks with 8 m s^−1^ speed in a square-shaped arena as a function of time. Dark purple corresponds to the DroneFlock’18 model for flying robots [43] based on ViSt; gold corresponds to the main model in this paper, the WillFull model. The motion provided by the WillFull model is more reliable: the most challenging situations (turning at corners) raise average density by only a factor of three and not six, as previously. Furthermore, these density peaks also became less frequent. The time average of the nearest neighbour distance is 22 ± 1.8 m for the WillFull model and 31 ± 4 m for the DroneFlock’18 model. Simulations were run for 500 agents in a 1400 m × 1400 m square arena, with an initial nearest neighbour distance of 20 m and an initial flock radius of roughly 300 m. Density is measured by dividing the arena into a 28 × 28 grid. *x*-axis, time; *y*-axis, maximum of the population of grids over the average population of non-empty grids. Thick line is smoothed from the original time series (thin line) with a co-moving averaging time window of 5 s.

With that measure, the WillFull model is compared with the best of our previous studies so far for moving artificial agents in confined environments [43]. We use the algorithm presented in [43] for comparison because no other analogous computations (i.e. simulations of realistic drone flocks) have been carried out (and, thus, it has rapidly become a standard reference in the field [64–66]). We shall use the expression DroneFlock’18 to denote this algorithm in the present paper. Both DroneFlock’18 and the algorithm introduced here are given the same realistic constraints on communication (80 m) and the same initial conditions and environmental parameters. The results for 8 m s^−1^ speed in figure 5 are reassuring: the WillFull model clearly outperforms the previous ViSt-based solution as it keeps the average density high but the maximal density relatively low, resulting in a much more reliable collective motion for a flock of 500 agents (for a visual comparison, see electronic supplementary material, videos S2 and S3).

**Figure 6.**
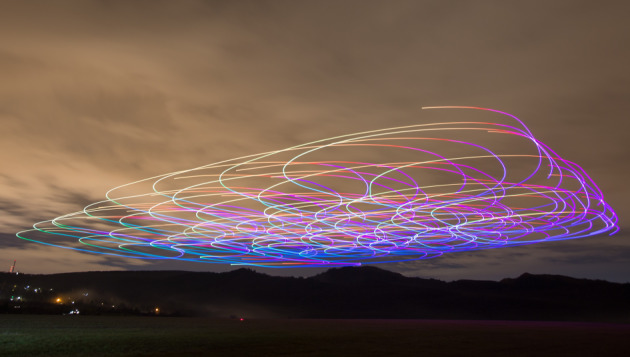
Long-exposure photograph of 52 aerial robots (drones) demonstrating the applicability of the WillFull model in real-life scenarios. Colour represents the flying direction of the agents. The virtual arena was a 200 m × 200 m square. Photo taken by Andras Tekus.

As a physically realized use case, we also applied the WillFull model to our tailor-made flock of outdoor quadcopters and successfully reproduced the above-mentioned simulated experiments in the real world with up to 52 drones at 8 m s^−1^ flocking speed in stable self-organization (figure 6). See electronic supplementary material, text S2 for drone-related technical details, figure S2 for density fluctuation analysis similar to figure 5, and video S4 for footage about the flights; or browse the interactive three-dimensional visualization of the flight logs at https://share.skybrush.io/s/pers-resp/.

The main reason for the WillFull model performing better than previous ones both in simulation (figure 5) and with real drones lies in the increased responsivity that facilitates quicker information propagation throughout the flock when there is an obstacle in the way. In previous models, frontal agents had the choice of stopping in front of a wall (and thus colliding with those behind) or continuing their motion in coherence with the rest of the flock (and thus colliding with the wall). Since information propagation was much slower, agent pressure accumulated much more at walls, which resulted in higher local density, potential collisions and slow and ineffective turns. To avoid collisions, the models had to be tuned for more stability and thus less optimal dynamics and for quite strong and long-ranging repulsion, which resulted in more spreading across the arena when not squeezed together by the walls. In the *will*ed model, information propagation is much faster; therefore, density fluctuations are convincingly reduced. Hence repulsion can be weaker, so oscillations inside the flock are less apparent. Therefore, friction does not have to dampen every difference in velocity, so changes can spread more quickly and the positive loop is complete. As a consequence, the scalability of the model to larger flock sizes is also substantially increased.

## 4. Discussion

Natural or robotic flocks perform well if they are capable of simultaneously maintaining coherent motion and changing their direction abruptly (the group as a whole) when a sudden external influence arises. Such strong impulses may correspond to an attack (by a predator) or confronting physical limits, e.g. walls, trees, etc. We aimed at understanding the complexity of the situation and constructed the simplest possible model that was able to optimize performance in terms of both kinds of behaviour.

The model we presented introduces a new kind of hierarchy into the set of interactions among the co-moving agents. This hierarchy corresponds to the degree by which an agent influences others, which is time dependent and is invoked by both internal and external conditions. The few parameters of this new type of hierarchy can be chosen in such a way that the flight of the flock optimally adapts to the environment, or to a particular decision of a member of the flock. Here optimal stands for smooth, collision-less (conflict-less) flight patterns. In general, we hypothesize that sophisticated designs of hierarchies are likely to result in new, efficient forms of collectively moving agents capable of processing complex information transmitted by the co-moving members of the group.

Our figures, especially figures 4 and 5, show the power of the concept introduced in this paper. For example, the left-hand side of the plot in figure 4 displays the expected trade-off (either performance or responsivity have relatively high values) for the most relevant existing models of flocking. However, our models, especially those combined with the spin-based model of Cavagna *et al.* [58], are able to be both ‘stable’ (coherent motion with nearly homogeneous density) and ‘sensitive’ (a sudden external influence does not destroy homogeneity). The corresponding symbols are in the region around the top right corner of the plot.

The main key of our concept is that external stimuli are assumed to trigger a new form of communication among flock members. The weight by which the ‘informed agents’ influence the trajectory of the rest of the flock is both simple and new. The contribution of the externally influenced members is increased, and without any new relevant information this additional feature gradually decreases. Thus, in our model the influence of the members of the flock changes with time as a function of the external conditions (and this is why we consider it to be a model of adaptive leadership). In this way new information about the environment can spread through the flock much faster than in prior models. We can look at the way in which our equations manifest themselves as follows: informed individuals give a special signal to others, and the others process this signal correspondingly. Our model works best if the range of interactions is such that the number of neighbours is above 10–20. For some species, and especially for robots, this can be easily realized—as seen in figure 6.

Since, in the framework we have presented, some individuals may play a decisive role, errors in sending information about the external perturbation can be a problem. A further, more complex question of intentional distortions [67,68] arises as well. Having avoided the persistence–responsivity trade-off agents might find themselves facing a new one, the speed–accuracy trade-off of information processing [69,70]. It is easy to think of prey species as those that are forced to choose speed over accuracy, hence they are exposed to frequent false-positive alerts [71], resulting in the spectacular overall patterns of murmurations. Questions of this sort are of great interest, and can be handled by suitable modifications to our approach. Further potential applications include a chase and escape situation where the information about the escaping agent spreads quickly in the flock, helping others to optimize their trajectory. We also believe that our model can be used efficiently as a collective control framework mostly in cases where adaptivity becomes a significant requirement, which is expected to happen more and more often in the future of artificial swarming.

## Supplementary Material

Electronic Supplementary Materials - Texts, Figures and Tables

## Supplementary Material

 Supplementary Video 1: Response simulations

## Supplementary Material

Supplementary Video 2: DroneFlock'18 model in confined environment

## Supplementary Material

Supplementary Video 3: WillFull model in confined environment

## Supplementary Material

Supplementary Video 4: WillFull model on real aerial robots

## Data accessibility

Simulation data are generated by the simulation framework published in [43,60], and can be reproduced using: https://github.com/csviragh/robotsim. Scripts to parse raw outcomes and the data illustrated in the article are available at: https://data.mendeley.com/datasets/3brbybg4nk/1.
