# Supplementary material for: Adaptive leadership overcomes persistence–responsivity trade-off in flocking
Source: J R Soc Interface. 2020 Jun 10;17(167):20190853. doi: 10.1098/rsif.2019.0853 (PMC7328404; doi:10.1098/rsif.2019.0853)
Supplement: Electronic Supplementary Materials - Texts, Figures and Tables [file rsif20190853supp1.pdf]

Supplementary material for the paper
Adaptive leadership overcomes persistence-responsivity tradeoff in flocking

Boldizsár Balázs[1], Gábor Vásárhelyi[2], and Tamás Vicsek[1]

[1]Eötvös Loránd University, Budapest, Hungary
[2]MTA-ELTE Statistical and Biological Physics Research Group, Budapest, Hungary

April 21, 2020

Text S1: Implementing the inertial spin model

The original equations of the inertial spin model introduced by Cavagna et al. [1] are:

$$\frac{d\mathbf{v}_i}{dt} = \frac{1}{\chi}\mathbf{s}_i \times \mathbf{v}_i$$

$$\frac{d\mathbf{s}_i}{dt} = \mathbf{v}_i \times \left[\frac{J}{v_0^2}\sum_{j\in N_i}\mathbf{v}_j - \frac{\eta}{v_0^2}\frac{d\mathbf{v}}{dt} + \frac{\xi}{v_0}\right]$$

The last term in the r.h.s of the spin time evolution is the noise, which is added to the desired velocity later in our simulations, so we omit that. The term before is responsible for the exponential decay of the spin if no new spin is given to the agent, so we wrote this decay explicitly in Eq. 3 of the main text with a free parameter τ, the characteristic lifetime of spin. The original model applied to fixed number of neighbours, but the number of neighbours changes in this paper and the results shall be comparable, thus the sum of the neighbours' velocity is normalised in Eq. 3. This is in accordance with the message of the original paper, namely that a certain quantity of spin is injected to the system by the turn-initialising agent. Without normalisation the quantity of the injected spin would depend on J and k, but with the average velocity taken into the equation it is uniquely described by J - as seen in Fig. 2 in the main text. Nevertheless one can always connect the couplings by $J^{original\ by\ Cavagna} = kJ^{applied\ in\ this\ paper}$. The rest of these equations gives back Eq. 3, if we measure both the spin s and the coupling J in the units of χ, what can always be done, because this inertial quantity is the essence what makes the model of Cavagna et al. the *inertial* spin model, hence its non-zero nature distinguishes it from those that resemble the self-propelled particle model of Vicsek et al. The first equation is an infinitesimal rotation what leads to tiny, finite rotations when one simulates in small time steps.

Text S2: Drone implementation technical details

The robotic framework in which we implemented the WillFull algorithm is exactly the same as detailed in [2]. We use middle sized (around 1.5 kg total takeoff weight) quadcopters based on the Pixhawk autopilot system, executing the ArduCopter software codebase. An Odroid-C1+ type companion computer is responsible onboard for running the swarm level control algorithm. The onboard computer runs Linux, the WillFull algorithm on it is implemented in C language. There is direct communication between the drones through an ad-hoc wifi network. Drones broadcast their status messages 10 times per second. The status messages contain the following pieces of information relevant to the WillFull algorithm: i) absolute timestamp; ii) geodetic position (latitude, longitude, altitude); iii) absolute velocity (x, y, z); iv) actual *will* value. Wireless

communication is broadcast type and thus gets local naturally due to the signal strength decay with space, which allows for scalability (see details on the communication network characteristics in [2]).

The arena in the drone experiments is predefined as a virtual GPS fence, i.e., drones are all aware of the specific geodetic coordinates of the walls (no active sensing is required to detect walls). However, in the software drones are restricted according to the WillFull model to react to walls only below a specific distance, so walls further than this distance are virtually unobservable by the drones directly.

Flight logs (including geodetic position) were collected by each drone individually onboard at 0.2s intervals. The interactive visualization of the flock flight at https://share.skybrush.io/s/pers-resp/ was created afterwards from the real flight logs resampled to the same timestamps. The coloring of the drones was not logged directly, therefore, the coloring of the visualization was recreated from the logged velocity directions.

Figure S1: Angular dependence of responses

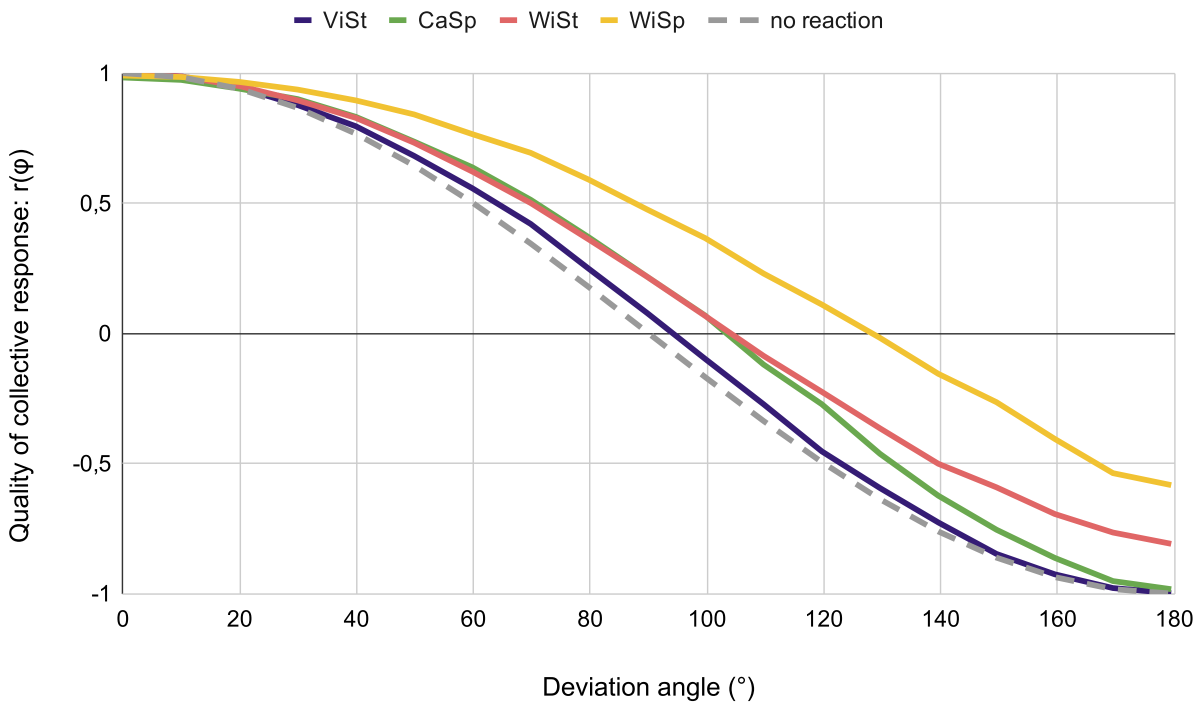

Figure S1: Quality of the collective response depending on the deviation angle, defined as $r(\varphi)$ in Eq. 10 of the main text. Number of topological neighbours is set to 15, number of agents is 2500. For comparison with Fig. 3 of the main text: $\mathrm{k}/\sqrt{N} = 0.3$. Averaging over angles these curves yield the following responsivity values (see Eq. 11 of the main text): ViSt (dark purple): 0.04, CaSp (green): 0.11, WiSt (red): 0.16, WiSp (gold): 0.36. Dashed grey line is a cosine curve, representing a flock with no reaction whatsoever to the deviation of agent 0, hence yielding R = 0.

Figure S2: Maximal density relative to average in real drone flights

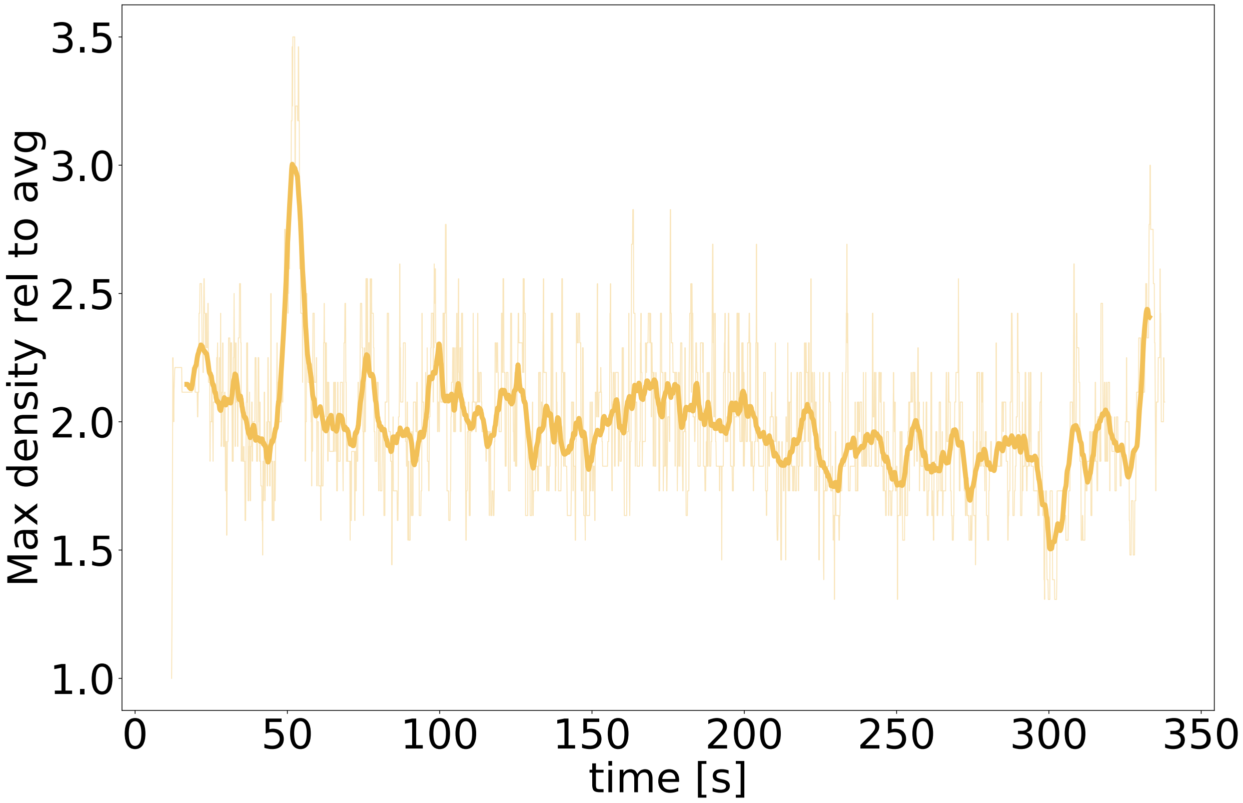

Figure S2: Maximum spatial density over average spatial density while 52 real drones flock according to the WillFull algorithm. Density is measured by dividing the 200 x 200 m arena into a 5 x 5 grid. Populated grids are occupied by 5.7 agents on time-average. Thick line is smoothed from the original time series (dim line) with a co-moving averaging time window of 5 sec. The figure resembles to Fig. 5 of the main text in that the curve moderately varies between 2 and 3. The reason for not having another curve for comparison highlights the advancement attained by the WillFull model: previous, overdamped models provide intolerable risk of mass-collision for 52 drones flying in real confined environments.

Table S1: Environmental parameters of simulations

Environment	Purified	Realistic
Fig. in main text	2, 3 and 4	5
$\sigma_\xi^2 [\mathrm{m}^2/\mathrm{s}^4]$	1, 1 and 2	0.2
$a^{max} [\mathrm{m}/\mathrm{s}^2]$	10	6
τ_c [s]	0.2	1
t_{delay} [s]	0.01	0.5

Table S1: Constraints on reaching the preferred velocity in the simulations as introduced in Eq. 8 of the main text

Table S2: Simulation model parameters

	DroneFlock'18	WillFull
$R_0^{repulsion}$[m]	50	34
$p^{repulsion}$ [1/s]	0.1	0.13
$R_0^{friction}$[m]	80	42
$C^{friction}$ [-]	0.5	0.13
$\Delta v_{ignored}^{friction}$ [m/s]	2	5.2
$p^{friction}$ [1/s]	1	4
$a^{friction}$ [m/s^2]	3	2
R^{Wall}[m]	30	50
$v^{wall-shill}$[m/s]	14	-
$p^{wall-shill}$[1/s]	2	-
$a^{wall-shill}$[m/s^2]	2.5	-
J^{spin} [-]	-	1400
τ^{spin} [s]	-	0.16
τ^{will} [s]	-	1

Table S2: Model parameters optimised by covariance matrix adaptation evolution strategy [3] for flocking speed of 8 m/s. Descriptions of parameters: [2]

Video S1: Comparison of flocking model responses to deviating leaders

In this video we compare the responses of the Couzin leader model, the Cavagna spin model and their will-based enhanced versions, the will standard model and will spin model in two scenarios: a) response of 1000 agents to a single leader in the middle of a flock deviating from the flock's direction by 90 degrees; b) response of 2500 agents to a single-leader-deviation of 120 degrees.

To visualize the response, we coded the color of agents according to their individual direction of motion. As can be seen, the responses of the willed models are much more intense in all cases.

Video also available online at https://youtu.be/6rbA-kmyADs

Video S2: Flocking simulation of 500 agents in a confined arena with the DroneFlock'18 model

In this video the DroneFlock'18 model [2] is used for a flocking simulation of 500 agents in a confined arena of 1400 x 1400 m, with flocking speed of 8 m/s, under realistic environmental conditions.

To visualize the response, we coded the color of agents according to their individual direction of motion.

As can be seen, collisions are avoided but the overall motion is not coherent enough and the density increases when the flock hits the corners.

Video also available online at https://youtu.be/QyYUEOOWf9E

Video S3: Flocking simulation of 500 agents in a confined arena with the WillFull model

In this video our new WillFull model is used for a flocking simulation of 500 agents in a confined arena of 1400 x 1400 m, with flocking speed of 8 m/s, under realistic environmental conditions.

To visualize the response, we coded the color of agents according to their individual direction of motion.

As can be seen, motion is much more coherent than in our previous attempts and there are no severe density fluctuations when the flock hits the corners, as collective decisions are driven by our concept of adaptive leadership.

Video also available online at https://youtu.be/12Xp0eig6Uk

Video S4: Self-organized flocking of 52 drones in a confined arena using the WillFull model

In this video we show a real use-case for the WillFull model: 52 quadcopters flocking inside 200 x 200 m virtual GPS walls at 8 m/s flocking speed.

The size of the arena is relatively small on purpose, to induce a high frequency of collective decisions at walls.

The most important message of this use-case is that the WillFull model and its concept of adaptive leadership can be used efficiently in practical applications to generate coherent and stable, yet very responsive motion for a large number of artificial agents.

Video also available online at https://youtu.be/87y3AsUOCaQ

Supplementary References

[1] Andrea Cavagna et al. "Flocking and Turning: a New Model for Self-organized Collective Motion". In: *Journal of Statistical Physics* 158.3 (Feb. 2015), pp. 601–627. ISSN: 0022-4715. DOI: 10.1007/s10955-014-1119-3. URL: http://link.springer.com/10.1007/s10955-014-1119-3.

[2] Gábor Vásárhelyi et al. "Optimized flocking of autonomous drones in confined environments". In: *Science Robotics* 3.20 (2018). ISSN: 24709476. DOI: 10.1126/scirobotics.aat3536.

[3] N. Hansen and A. Ostermeier. *Completely derandomized self-adaptation in evolution strategies.* 2001. DOI: 10.1162/106365601750190398.
